# GPs’ perspectives on eHealth use in the Czech Republic: a cross-sectional mixed-design survey study

**DOI:** 10.3399/bjgpopen19X101655

**Published:** 2019-07-24

**Authors:** Adam Klocek, Martina Šmahelová, Lenka Knapová, Steriani Elavsky

**Affiliations:** 1 PhD Student, Faculty of Social Studies, Masaryk University, Brno, Czech Republic; 2 Academic Researcher, Faculty of Social Studies, Masaryk University, Brno, Czech Republic; 3 PhD Student, Faculty of Informatics, Masaryk University, Brno, Czech Republic; 4 PhD Student, Faculty of Education, University of Ostrava, Ostrava, Czech Republic; 5 Academic Researcher, Faculty of Social Studies, Masaryk University, Brno, Czech Republic

**Keywords:** clinicians, eHealth, general practice, ICT, mHealth, telemedicine

## Abstract

**Background:**

Digitalisation of health services is among the top priorities of the European Union (EU), yet take-up of eHealth tools is slower in some EU countries than others.

**Aim:**

The aim of this study was to evaluate the use of information communication technology (ICT) and eHealth tools by Czech GPs, to elucidate their motivation and barriers to the adoption of eHealth technologies.

**Design & setting:**

A cross-sectional, mixed-design survey study, administered online and conducted with GPs from seven randomly selected Czech regions. Of the invited 777 GPs, 196 participants responded (25% response rate) and 153 completed the survey.

**Method:**

Quantitative (measured using, for example, the eHealth readiness scale) and qualitative (thematic analysis) methods were used.

**Results:**

Hierarchical multilinear regression (controling for age, sex, and city size) showed that ICT usage in general practice was predicted by eHealth readiness. Among GPs with their own practice, age and practice size also predicted ICT use. Analysis of barriers specific to mobile health tools identified obstacles on the side of GPs (such as low perceived usefulness), patients (such as lack of interest), and contextual barriers (such as lack of time).

**Conclusion:**

In addition to system-level change, educating Czech GPs about the benefits of eHealth tools for better patient–provider interaction and quality of care is necessary to facilitate eHealth adoption and usage in the Czech Republic.

## How this fits in

Czech GPs use ICT tools primarily for communicating with patients rather than for providing care.

Use of ICT tools for health in general practice (in other words, eHealth use) was predicted by lower age, larger practice, and higher eHealth readiness.

GP education should focus on eHealth tools for intervention and care quality.

## Introduction

eHealth is a fast-developing field that refers to the organisation of health services using the internet and related technologies.^1,2^ The promise of eHealth lies in reducing cost of care and medical errors^3^ while improving healthcare delivery and access to services.^4^ It is also vital for increasing diagnostic accuracy, improving decision-making, and facilitating the design and delivery of tailored or complex treatments.^4,5^ Across the globe, countries are making investments in setting up electronic health records, telemedicine, and IT infrastructures as part of digitalisation of health care, with the expectation that, by 2020, 90% of clinicians will be making decisions based on some eHealth system providing the most suitable data.^6^

The EU has formed strategies (such as EU 2020) to embrace eHealth in general practice.^4–6^ Despite the common strategic goals, differences in organisational healthcare system factors across EU member states require eHealth adoption efforts to reflect the unique needs of stakeholders.^7^ In the Czech Republic, an EU member country, the strategies to embrace eHealth in general practice have been focused on organisational changes in medical law (such as the Act on People’s Healthcare) and the implementation of healthcare system digitalisation (electronic health records or ePrescription programs), with the first attempts at formulating an eHealth strategy dating back to 2007.^8^

Within the Czech healthcare system, which offers universal healthcare coverage, GPs provide primary care and recommend specialised care when needed, but the core of their work lies in preventive care, providing diagnosis and treatment for a wide range of health problems; organisational work to run their practice; and interacting and communicating with their patients.^9^ Although the use of eHealth and other ICT can significantly ease some of these processes, its uptake by GPs has been modest. Although most general practices now use computers (96.1% in 2015),^4^ specific uses vary substantially: use for electronic transaction of patient data increased from 24.7% (2009)^10^ to 33% (2014);^7^ electronic storage of patient data from 67.7% (2009)^10^ to 84.5% (2015);^4^ and, most importantly, ePrescriptions, which was forced into effect by a change in medical law in January 2018, increased from 0% (2009)^10^ to 87.21% (2015).^4^ Although 39.27% of GPs also use ICT for making appointments,^4^ the bulk of ICT use within Czech general practice remains associated with administrative tasks (such as submitting insurance claims or filling in forms) instead of provision of care. Efforts to implement national electronic health record system have stalled (project suspended in 2012 due to financial, legal, and political problems) in the same year as the Czech National eHealth Centre in Palacky University Olomouc (NTMC) was established. There are no systematic data on the adoption rates of different aspects of eHealth in the Czech Republic and there is limited information on factors that could facilitate faster adoption of ICT and eHealth tools in the context of clinical care in the Czech Republic.

In a recent review, Yusif *et al* ^11^ point out that ICT readiness (at the individual, community, and organisational levels) is generally among the key factors in ICT adoption. This is in line with Bronfenbrenner’s ecological systems theory,^12^ which hypothesises that factors at multiple levels interact to influence behaviour. With respect to eHealth technology, for example, aspects of the national healthcare system, the country’s legal system (that is, macro- and exo-system level factors) could shape eHealth use and readiness rates.^3^ At the same time, at the meso- and micro-system levels, specific characteristics of GPs’ practices and patient populations may play a role (such as, city size, practice size, access to ICT, and access to eHealth education). For example, one might expect lower eHealth use among GPs serving older patients, in smaller practices, or in practices located in rural areas.^4^ These associations could be explained by the generally lower exposure to ICT and fewer resources to incorporate new technologies into daily clinical practice,^4^ or by the lack of cooperation between primary care clinicians, academics, regional and governmental policies, and technical specialists.^7^ Finally, at the individual level, eHealth use may also be influenced by individual characteristics of GPs, as well as their preferences, attitudes, intentions, and motivations.^3^ Slow adoption of eHealth technology can be due to the lack of practitioner (and/or patient) awareness of eHealth technologies and their benefits in general.^13^ Factors such as professional burnout may also impact eHealth use; for example, usage of eHealth tools (such as electronic health records and electronic order entry) was related to lower satisfaction and increased risk for physicians’ burnout in a national study of US physicians, possibly due to potential information overload, changes in the system of work, and/or added tasks necessary for daily practice.^14^ This evidence of association between eHealth usage and higher burnout was also supported in the context of advanced practice nurses, with insufficient time for documentation entry being the main contributing factor.^15^

In spite of the increasing trend of eHealth technology adoption across Europe, Czech GPs remain overall more negatively inclined and their eHealth usage is lower as compared to their counterparts in other parts of Europe.^4^ Most of the existing literature on explaining eHealth usage also focuses on healthcare contexts with relatively high adoption of eHealth tools (so-called ‘frontrunner’ countries).^8,11^ Providing diverse data from contexts with lower overall usage or slower adoption rates would enhance understanding of the key facilitators and barriers to eHealth adoption in clinical practice. In terms of the Czech Republic, there are also no publicly available data on the use of mobile health technologies, in particular, as a specific subtype of eHealth technology reflecting the latest technological trends within the general practice context. This mixed-design study therefore assesses ICT usage as well as attitudes towards eHealth and mobile technology usage within general practice to identify key factors related to eHealth use and eHealth readiness among Czech GPs. The specific aims of this study were to (1) describe patterns of ICT use and attitudes towards eHealth use among GPs in the Czech Republic; (2) identify factors explaining ICT use and eHealth readiness of GPs in the Czech Republic; and (3) assess barriers to mobile health technology among GPs in the Czech Republic.

## Method

### Participants

To recruit participants, available online databases of Czech GPs were scanned (*www.znamylekar.cz*, Czech equivalent to *www.docplanner.com*, which contains contact information for more than 2000 GPs)^16^ and seven out of 14 regions of the Czech Republic were randomly selected. Listings of all GPs and their contact information were then downloaded into a single database. All potential participants with reported email contact (*n =* 777) were sent an invitation via email to participate in an online survey, (accounting for 15% of all GPs in Czech Republic, out of a total of 5121).^17^

Additionally, all potential participants with a reported phone number (*n* = 527) were contacted by telephone (including those already contacted by email). The GPs were contacted again 2–4 weeks later and a reminder about the survey termination date was included. All participants were invited to forward invitations to their colleagues (the chain-referral sampling method). In the end, 196 participants initiated the survey (overall response rate of 25%). Of the total number, 43 participants were excluded for various reasons (either provided no data, opened survey but did not continue, or were not GPs). This resulted in a sample of 153 participants to be included in the analyses. The data selection process lasted from October 2017–January 2018.

### Study procedures

All participants were presented with an informed consent statement on the first page of the online survey. Completion of the survey signified consent to participate. The following subheadings describe the information that was assessed as part of the survey.

#### Demographics

Several demographic questions were asked: sex, age, city size (population), and period of working as a GP. Questions regarding practice ownership (duration of practice, size of practice) were also included.

#### eHealth use in general practice

Given the limited scope of applications of eHealth tools within the Czech healthcare system (for example, no universal electronic health records), eHealth use was assessed in this survey through six yes/no (scored 1/0) questions about ‘ICT use in general practice’. Subsequently, the ‘eHealth use in general practice’ variable was aggregated as the sum of the following dichotomous items: (1) website usage in general practice; (2) social network or Facebook usage in general practice; (3) ICT use (via SMS, email, or web-portal) in scheduling of patients in general practice; (4) ICT type and availability (the use or presence of TV, tablet, or wifi) in a GP waiting room; (5) ICT use (via SMS, email, or web-portal) in communication with patients in general practice; and (6) sharing of results with patients through ICT (via SMS, email, or web-portal). The resulting score range for ICT use in GP was from 0–6 points.

#### eHealth readiness

The eHealth Readiness Scale^18^ is a 7-item, 6-point Likert-type scale used to assess preparedness to use health-promoting technologies connected to the internet. Authors of the scale reported a stable one-factor structure with good internal reliability on two samples (Cronbach’s *α* = 0.86 for students, *α* = 0.83 for mental health providers). In the present study, the scale was adapted for the GP environment, reporting internal consistency of Cronbach *α* = 0.86.

#### Barriers

Barriers to using mobile technologies in GP were assessed using the open-ended question: *‘What prevents you from using mobile technologies while working with patients?*’. One-hundred and ten answers to the open question about barriers were received, two of which were not possible to code due to their brevity and unclear meaning. Principles of summative content analyses were applied,^19^ and a total of 108 open answers from the responders were analysed. Some responders acknowledged more than one barrier. The analysis was conducted by two coders (AK, MŠ), who conducted coding independently. Both coders have experience in qualitative analysis. The primary in vivo codes were organised into themes and categories by a sequence of abstraction steps. Discrepancies were discussed with the whole team and resolved by mutual discussion and agreement.

#### Control variable*s*

The analyses controlled for indicators of personal ICT use: ICT device ownership (that is, the number of ICT devices owned by GPs at home, outside of their practice) and self-reported digital skills (one item 5-point Likert-type scale from 1 ‘very bad’‘ to 5 ‘very good’). Additionally, professional burnout was assessed using the Physician Burnout Survey questions.^20^ The first five items from a 6-point Likert-type scale were used and adapted to the GP environment, reporting Cronbach’s *α* = 0.77.

The entire survey was administered using the Qualtrics online platform. The survey was anonymous and restricted to one user completion from a single IP address. All administered measures not available in Czech were translated into Czech language using the method of reverse translation^21,22^ by independent translators, with inconsistencies being reconciled by the author team, which included a bilingual speaker.

### Analysis

SPSS (version 25) was used to analyse the data. Descriptive statistics were computed, followed by bivariate correlations and hierarchical linear regression analyses. Sex differences and practice ownership/non-ownership differences were analysed using independent sample *t*-tests. Hierarchical linear regression analysis was conducted to predict ICT use in general practice. Block 1 represented control variables (age, sex, size of city, professional burnout). Block 2 represented potential predictors of ICT usage in general practice (number of personal ICT devices owned by the GP, digital skills, and eHealth readiness). The same regression was repeated in a subsample of GPs who owned their own practice, with size of practice as an additional predictor in Block 1. Assumptions for multiple linear regression were tested.

## Results

### Descriptive characteristics and eHealth usage in general practice

The descriptive statistics for the whole sample are available in Table 1. The pattern of reported ICT use can be seen in Table 2. Two-thirds of GPs (*n* = 95; 62.1%) perceived using mobile technologies (SMS, smartphone, tablet, and other devices) in the general practice as useful, and endorsed a range of uses (see Table 2). GPs that owned their own practice (*n* = 99, 65.1%) were older (mean age [SD] = 50.93 [12.2] versus 35.11 [10.15]; *t*_144_= 7.712, *P* = 0.000) and more commonly practiced in smaller towns of <20 000 inhabitants (50.5% versus 10.1%, χ^2^=16.753, *P*<0.01). Although GPs with their own practice personally owned more digital devices (mean [SD] =3.40 [1.08] versus 2.79 [1.39]; *t*_144_= 3.004, *P*<0.01), they perceived their ICT skills as lower (mean [SD]=3.60 [0.93] versus 3.94 [0.87], *t*_144_=–2.116, *P*<0.05), and reported lower eHealth readiness (mean [SD] = 25.16 [7.70] versus 28.15 [6.27]; *t*_144_= -2.294, *P*<0.05).

**Table 1.** Descriptive characteristics of responding Czech GPs (*n* = 153; 2017–2018)

	Responses, *n*	Mean+standard error	Standard deviation	Range (min–max)
**Age**	147	46.03+1.145	13.883	57 (26–83)
**Sex**	146	65.4% female		1 (1–2)
**City size**	146	4.11+.133	1.611	5 (1–6)
0-999 inhabitants		2.7%		
1000-4999 inhabitants		17.60%		
5000-19999 inhabitants		21.60%		
20 000-99 999 inhabitants		15.50%		
100 000-299 999 inhabitants		10.10%		
≥300 000 inhabitants		32.40%		
**Years of practice**	152	15.839+1.08	13.352	57.5 (0.5–58)
**Own practice**	99	64.70%		1 (1–2)
**Years of own practice**	99	15.116+1.013	10.075	39 (1–40)
**Number of patients in own practice**	99	1901.4+61.419	608.017	3910(990–4900)
**ICT use in general practice (aggregated)**	153	2.98+0.152	1.879	6 (0–6)
**Number of ICT devices owned by GP**	147	3.30+0087	1.05	4 (2–6)
**Self-reported ICT skill**	147	3.69+0.076	0.926	4 (1–5)
**Professional burnout syndrome**	147	16.02+0.257	3.117	20 (5–25)
**eHealth readiness**	142	26.05+0.623	7.42	32 (10–42)

**Table 2.** Descriptive characteristics of eHealth usage by responding Czech GPs (*n* = 153; 2017–2018)

	ICT used	Frequency, %
Owned technology	TV	23.5
	Wi-Fi	24.2
	Camera/e-evidence of patients	5.9
	Tablet	1.3
Communication with patients	Telephone	98.7
	SMS	41.8
	Email	73.2
Sharing results of laboratory assessments	Patient calls the practice	96.1
	The practice calls the patient	73.2
	Results sent via SMS	18.3
	Results sent via email	48.4
	Results sent via web-portal	3.3
	Results sent via post-office (hard copies)	11.8
Using mobile technologies in general practice	‘It seems useful…’	62.1
	for making appointments	76.5
	for sending results of assessments	65.4
	for monitoring patients’ health outcomes	30.7
	for answering patients’ questions	48.4
	for monitoring of medication	35.3
	for motivating patients towards a more healthy lifestyle	29.4
	for showing care and interest in patients’ health problems	28.8
	for monitoring and feedback about health status (e.g. blood pressure)	41.8
GPs´ with their own practice (*n* = 99) public internet profile	Own website	53.5
	Planning to have website	9.1
	Social media	14.1
	Planning to have social media	3.0
Setting up appointments	Personal communication	76.8
	Telephone	87.9
	Email	51.5
	SMS	17.2
	Web-portal	13.1
	No ICT: served on first-come-first-served basis	51.5

### Factors associated with eHealth usage in general practice

Bivariate associations among all variables are reported in Table 3. The results of the hierarchical regression analyses are presented in Tables 4 and 5. Among the entire sample, ICT use in general practice was uniquely associated only with eHealth readiness (*β* = 0.381, *P* = 0.000), with the overall model accounting for 20.1% of the total variance in ICT use in general practice *F* (7,132) = 4.736, *P*<0.001). In a subsample of GPs who own their practice, ICT use in general practice was uniquely associated with age (*β* = -0.328, *P*<0.01), practice size (*β* = 0.227, *P*<0.05), and eHealth readiness (*β* = 0.343, *P*<0.01). The overall model accounted for 40.4% of the total variance in ICT use in general practice (*F* [8,83] = 7.023, *P*<0.000).

**Table 3.** Results of bivariate correlation coefficients among responding Czech GPs (*n* = 153; 2017–2018)

	1	2	3	4	5	6	7	8	9	10
1. Age	–	-0.023	0.924^a^	0.053	0.003	-0.306^a^	-0.310	0.036	-0.229^a^	0.052
2. City size	0.220^b^	–	-0.045	-0.131	0.010	-0.095	-0.061	-0.033	0.029	0.154
3. Years of practice	0.905^a^	-0.045	–	0.069	-0.008	-0.320^a^	-0.291^a^	0.122	-0.206^b^	0.019
4. Practice size	0.053	-0.131	0.069	–	NA	NA	NA	NA	NA	NA
5. Burnout	0.122	-0.023	0.097	-0.023	–	0.051	0.042	-0.006	0.005	-0.071
6. eHealth readiness	-0.256^b^	-0.198	-0.300^a^	0.342^a^	0.079	–	0.428^a^	0.263^a^	0.406^a^	-0.176^b^
7. ICT skill	-0.278^a^	-0.129	-0.260^a^	0.225	-0.076	0.418^a^	–	0.373^b^	0.155	-0.092
8. Number of owned ICT devices	-0.030	0.010	-0.049	0.343^a^	0.038	0.326^a^	0.360^a^	–	0.189^b^	0.017
9. ICT usage in general practice	-0.370^a^	-0.105	-0.358^a^	0.342^a^	-0.028	0.522^a^	0.257^b^	0.290^a^	–	-0.048
10. Interest in mHealth usage	-0.036	0.191	-0.005	-0.208^b^	-0.184	-0.165	-0.064	-0.031	-0.092	–

^a^ *P*<0.01. ^b^ *P*<0.05. Correlations above the diagonal refer to associations in the entire sample. Correlations below the diagonal refer to associations observed in GPs who own their practice.

**Table 4.** Results of hierarchical multilinear regression estimating the association between ICT usage in general practice and its predictors among responding GPs (*n* = 153; 2017–2018)

	Model	R^2^ change	β	Standard error	Critical value	Significance
1	Age	0.050	-0.224	0.011	-2.662	0.009
	Sex	0.021	-0.144	0.340	-1.654	0.100
	City size	0.004	0.061	0.098	0.712	0.478
	Burnout	0.004	0.061	0.051	0.730	0.467
		0.066		*F* (4,135)	2.397	0.053
2	Age	0.020	-0.143	0.012	-1.651	0.101
	Sex	0.009	-0.097	0.324	-1.166	0.246
	City size	0.007	0.085	0.092	1.052	0.295
	Burnout	0.002	0.047	0.048	0.602	0.548
	Digital skills	0.014	-0.118	0.185	-1.259	0.210
	Digital devices	0.011	0.105	0.151	1.209	0.229
	eHealth readiness	0.145	0.381	0.022	4.268	0.000
		0.134		*F* (3,132)	7.400	0.000

**Table 5.** Results of hierarchical multilinear regression estimating the association between ICT usage in general practice and its predictors among responding GPs with their own practice (*n* = 99; 2017–2018)

	Model	R^2^ change	β	Standard error	Critical value	Significance
1	Age	0.172	-0.415	0.013	-4.455	0.000
	Sex	0.026	-0.161	0.336	-1.685	0.096
	City size	0.006	0.080	0.105	0.812	0.419
	Practice size	0.138	0.372	0.000	4.064	0.000
	Burnout	0.003	0.055	0.050	0.603	0.548
		0.305		*F* (5,86)	7.556	0.000
2	Age	0.107	-0.328	0.013	-3.473	0.001
	Sex	0.008	-0.090	0.328	-0.970	0.335
	City size	0.006	0.079	0.100	0.841	0.403
	Practice size	0.052	0.227	0.000	2.357	0.021
	Burnout	0.000	0.021	0.047	0.243	0.809
	Digital skills	0.003	-0.058	0.185	-0.582	0.562
	Digital devices	0.010	0.099	0.158	1.014	0.314
	eHealth readiness	0.118	0.343	0.023	3.310	0.001
		0.098		*F* (3,83)	4.568	0.005

### Barriers to mobile technology usage in general practice

These analyses revealed three main themes linked to barriers of technology usage in the GP setting: barriers on the side of GPs (*n* = 28, 26% of all reported barriers), barriers on the side of patients (*n* = 35, 32%), and contextual barriers (*n* = 64 , 59%). Six responders reported that they did not perceive any barriers for usage of mobile technologies.

#### Barriers on the side of GPs

Barriers on the side of GPs were related to three main subthemes. First, GPs talked about their prejudices against mobile technologies. They did not perceive mobile technologies as useful in direct work with patients, and talked about these technologies as useless or even unsafe. One responder commented:

*‘TV and internet are harmful — people live in a virtual reality and it reduces their intellect*.’ (ID32)

Second, when GPs perceived their work as primarily related to direct work with patients, they mentioned a preference for face-to-face communication. Some of the GPs sharing this concern also expressed their fear of losing personal contact with patients, which is, according to them, already limited. Personal factors, mainly the age of GPs or their perceived lack of digital skills, were also mentioned as barriers on the side of GPs.

#### Barriers on the side of patients

Barriers on the side of patients were also related to three subthemes. GPs considered patients' lack of ICT knowledge and hardware (such as smartphones and tablets) as the main barrier. This barrier was mainly linked to older patients. Lack of interest was also mentioned, when GPs perceived mobile interventions as unattractive to patients. One responder commented:

‘*I see the potential reluctance on the side of the patient as an obstacle, as it is a relatively large interference with their privacy*.’ (ID195)

They also mentioned poor awareness about the mobile interventions being used, which could also be linked with patients’ lack of interest. The third subtheme was related to a concern about misusing existing mobile technologies on the side of the patients. Linking this with mobile technologies already used (for example, for communication with patients), the GPs perceived that patients were using them in an excessive manner or in inappropriate situations.

#### Contextual barriers

Contextual barriers were linked with barriers on the side of the existing healthcare system. Lack of time was identified as the main barrier, with GPs perceiving their profession as already full of administrative work with a lack of staff to help them. Some mentioned the perception that a substantial amount of time was needed to implement a mobile intervention and subsequently also for interpretation of the results. Additionally, barriers linked with technical background were mentioned (not having hardware or technical support, and lack of knowledge about any mobile interventions suitable for the Czech context ready to be used), as one responder mentioned:

*‘So far, this type of monitoring is not available, I do not know of any application that would enable this while also protecting personal data*.’ (ID36)

Fear of inadequate security of patients' personal information and GPs’ ability to secure them properly was also mentioned by others along with concerns about incompatibility of mobile interventions with existing medical software that GPs already use in practice. Financial aspects represented the third barrier; some GPs mentioned that they do not have the finances to buy new mobile technologies and interventions, or they perceived the absence of payment for mobile interventions by insurance companies as the problem.

## Discussion

### Summary

This study identified the predictors and barriers of eHealth and ICT usage in a sample of GPs in the Czech Republic. At the individual level, eHealth readiness was identified as an important factor in ICT use among Czech GPs. An association was also found between GPs’age and their ICT usage in general practice, but this association was significant only in the subsample of GPs who owned their practice. The main barriers specifically to mobile health technology (mHealth) usage in general practice were contextual (lack of time, technical background, support); on the side of patients (lack of knowledge, interest); and on the side of GPs (prejudice, lack of skills, preference for face-to-face communication). A relatively small number of GPs mentioned financial barriers, but GPs reported prejudices about mHealth and they assumed these prejudices on the side of their patients as well (especially older patients). GPs expressed the view that there are limited options for mHealth deployment in the Czech context, citing not being aware of any tools available for immediate use in clinical practice. While the lack of time was the main reported barrier, the GPs expressed that system or organisational level change may be required to provide them with the time and/or incentives needed to deploy mHealth tools more broadly. All in all, however, these results indicate that resources per se do not sufficiently explain the low eHealth usage among Czech GPs, as they have both access to and experience with ICT. Instead, factors such as eHealth readiness and age, at the individual level, and practice size, at the micro- and meso- levels, may drive eHealth adoption in the Czech general practice context.

### Comparison with existing literature

Identifying eHealth readiness as an important predictor of eHealth use is in line with other research across the world,^11,23–25^ but this factor has not been assessed among Czech GPs before. The observed association between GPs’ age and their ICT usage in general practice is to be expected, given the fact that practice ownership is more likely in older GPs who may not engage ICT within their practice to the same extent as younger GPs.^26^ Indeed, younger age was associated with more engagement with eHealth tools in a large survey of European GPs in a recent study.^27^ In the present study, GPs with their own practice also perceived their ICT skills to be poorer and their eHealth readiness was lower than that of other GPs, suggesting that, with increasing age, these factors may take on more importance in terms of eHealth use.^28^ Interestingly, in the study by Torrent-Sellens *et al,* ^27^ self-employed physicians showed more propensity towards eHealth usage, warranting further investigation of how factors such as age, self-employment status, and practice size interact to influence uptake of eHealth. Additionally, the current study found that the greater the number of patients, the more ICT was used in the practice. All of this is in line with the study by Decker *et al,* ^29^ which also found associations between older age, practice size, and the adoption of electronic health record systems. There are likely several reasons that underlie the association between age, size of practice, and ICT use First, from a pragmatic standpoint, the daily operations of larger practices may require more intensive deployment of ICT to manage caring for a greater number of patients, similar to commercial enterprises.^30^ It is also possible that larger practices have more resources or capital to invest into new ICT tools as compared to small ’family’ practices. Another explanation could be the increasing diversity among patients that accompanies larger patient numbers.^31^ Engaging ICT tools may thus be a response to the diverse demands of the various patient subpopulations, especially younger patients. It is reasonable to assume that the trend in the adoption of ICT within general practice will continue to increase as younger generations of patients who use these technologies age.

With respect to attitudes towards mHealth use more specifically, the findings were positive in that two-thirds of GPs saw mobile technologies in medical context as useful. Furthermore, a substantial portion of the GPs (41.8%) indicated they would use an application capable of monitoring health status and behaviour simultaneously along with providing feedback to a physician. This is encouraging given the rising emphasis on behavioural management as part of both primary and secondary prevention efforts, aligning with the trend in mHealth usage for monitoring (telemedicine) and behaviour modification through prescription-based health mobile apps.^32^

Still, it would be of importance (as part of educational efforts, for example) to address the somewhat ageist views observed among the GPs in terms of older adults and ICT; that is, GPs reported lower use of ICT by older adults and expressed, in the qualitative analysis, that older patients would not be interested in, willing to use, or capable of using technology in their interactions with GPs or as part of primary care. Although this may be true for some older adults, especially in older ages (above 75),^33^ there is evidence that older adults are interested in engaging with new technology if it is perceived as useful.^34^

Interestingly, in the GPs’ responses about barriers to mHealth usage, the cited barriers relating to perceived lack of time appeared to be associated with the GPs’ preference for face-to-face communication and their perception that it is already reduced by complicated administration procedures which restrict time for patients in the practice. From a quality of care perspective, however, mHealth tools can be used effectively to enhance the quality of personal interactions with patients.^32^ Thus, reframing perceptions of barriers into opportunities for continuous quality improvement of care and patient satisfaction should be an integral part of intervention efforts to increase eHealth or mHealth usage in clinical care.^35^ Notably, another type of barrier cited was related to the concerns regarding security of the data obtained through mHealth tools. The present survey took part during the adoption of the EU legislation related to the General Data Protection Regulation (GDPR), a fact that could have inflated the GPs’ concerns over data safety during the sampling phase of this research.

### Strengths and limitations

This is the first study that examined eHealth and ICT usage among Czech GPs. Although sufficient in statistical power for the tests performed, the sample size in this study was relatively small, with a response rate of 25%. However, this is in line with non-incentivised online survey response rates.^36,37^ In addition, online databases were sampled sampled from only seven out of 14 regions of the Czech Republic. The findings may therefore reflect attitudes of a self-selected group of participants who may have been either positively or negatively motivated to respond to the survey. Some variables (practice size) were assessed only among GPs who owned their own practice, and associated findings should be interpreted in that context.

The cross-sectional nature of the study does not allow for making causal inferences, but the data on previously unevaluated predictors of eHealth use within the Czech context (such as eHealth readiness and professional burnout) serve as the first stepping stone in future systematic efforts to track eHealth adoption rates and its predictors.

### Implications for research and practice

Future studies are needed on larger and representative samples of GPs from a more diverse set of European countries to distinguish culture- or country-specific needs (for example, with respect to a particular system-level change or eHealth component) from universal themes related to eHealth implementation (such as data security). In the Czech Republic, more specifically, studies should aim to obtain samples representative of all 14 regions, with a specific focus on oversampling GPs from smaller practices or those without internet resources. Ideally, such studies would track eHealth adoption rates over time to help characterise GPs behaviour with respect to eHealth adoption, given how fast ICTs develop. The authors suggest that future research should incorporate other variables potentially affecting eHealth adoption in the GP context at all levels of influence (individual, micro, meso, exo, and macro).^12^ Consequently, intervention efforts to enhance eHealth readiness and eHealth usage among Czech GPs should be deployed at each of the levels or across multiple levels of influence. Educational programmes targeting Czech GPs who underutilise eHealth tools may serve as one starting point and should supplement system-level changes. Moreover, future surveys incorporating the perspectives of all stakeholders (GPs, patients, and policy- and decision-makers) appended by qualitative data are needed as well.

These findings have also implications for professional preparation of GPs and the continuing education context. Although system-level change is often pursued first, arguably because it produces more immediate impact on changing practices of GPs in clinical care (such as was the case with legally mandating ePrescriptions in the Czech Republic),^38^ these results indicate that it may be advantageous to, at the same time, pursue education efforts about the benefits of eHealth tools to motivate GPs to incorporate ICT within their practice. In this way, enhancing eHealth readiness of GPs may also serve to increase the chances that any subsequent system change will be efficiently used and generally accepted.^39^

Using macro-level approaches, such as legislating healthcare system changes (as employed in ePrescription case), should be accompanied by educating and motivating GPs to utilise eHealth tools. Intervention programmes should focus on the discussion of the advantages of eHealth tools not only in terms of improving administrative efficiency within general practice but primarily in terms of facilitating patients’ health behaviour changes, improving patient–provider interactions, and improving quality of care. Interventions targeting older GPs and smaller practices, where motivation and education have been most lacking, may be particularly needed to enhance eHealth literacy and use in the Czech Republic and so ensure alignment with EU strategic goals and global trends in healthcare delivery.
